# Framing the fallibility of Computer-Aided Detection aids cancer detection

**DOI:** 10.1186/s41235-023-00485-y

**Published:** 2023-05-24

**Authors:** Melina A. Kunar, Derrick G. Watson

**Affiliations:** Department of Psychology, The University of Warwick, Coventry, CV4 7AL UK

**Keywords:** Mammogram, Artificial Intelligence, Visual search, Computer-Aided Detection (CAD), Over-reliance, Framing

## Abstract

Computer-Aided Detection (CAD) has been proposed to help operators search for cancers in mammograms. Previous studies have found that although accurate CAD leads to an improvement in cancer detection, inaccurate CAD leads to an increase in both missed cancers and false alarms. This is known as the over-reliance effect. We investigated whether providing framing statements of CAD fallibility could keep the benefits of CAD while reducing over-reliance. In Experiment 1, participants were told about the benefits or costs of CAD, prior to the experiment. Experiment 2 was similar, except that participants were given a stronger warning and instruction set in relation to the costs of CAD. The results showed that although there was no effect of framing in Experiment 1, a stronger message in Experiment 2 led to a reduction in the over-reliance effect. A similar result was found in Experiment 3 where the target had a lower prevalence. The results show that although the presence of CAD can result in over-reliance on the technology, these effects can be mitigated by framing and instruction sets in relation to CAD fallibility.

## Public significance statement

There has been substantial recent investment into Artificial Intelligence and Machine Learning Algorithms to develop Computer-Aided Detection (CAD) for tasks such as searching for cancers in mammography. However, although there is benefit to using CAD, its presence also leads to readers becoming over-reliant on the technology. This research shows that prior knowledge about the costs of CAD can reduce this over-dependence, while still retaining its benefits. The research is important for understanding how humans interact with CAD technology, for optimal medical screening behaviour.

## Introduction

Visual search is an important part of our daily life. For example, we may search for a car in a car park, a face in a crowd or a set of keys on a cluttered desk. Visual search tasks are also important for some critical applied tasks that relate to health and safety. For example, airport security personnel search images of bags for prohibited items and medical health care professionals search medical images for indications of cancers. Missing a target (prohibited items or cancers) in these latter search tasks can result in harmful outcomes. Therefore, it is important that in these tasks, people can search and find their target accurately and efficiently.

In breast cancer screening, visual search has been shown to be complex and can result in a failure to find a high proportion of cancerous indicators (e.g. Evans et al., 2013a, 2013b). To minimise these errors, different procedures have been implemented to try and reduce miss errors and optimise search. For example, in the UK each mammogram is viewed or ‘read’ by two readers, and any disagreement is then considered at arbitration. The double reading procedure is effective and has been found to increase cancer detection and reduce the proportion of missed cancers (e.g. Kunar et al., 2021; Taylor & Potts, 2008). However, with the increase in the number of women needed to be screened, and a decrease in the number of trained radiologists, this procedure may not be future-proof (James et al., 2010). Instead, other ways to improve mammography reading need to be considered.

The use of Artificial Intelligence (AI) and Machine Learning has been proposed as one approach to improve medical screening. Computer-Aided Detection (CAD) has been recommended as a ‘virtual’ second reader where computer algorithms are used to highlight ‘suspicious’ areas in a mammogram for a human reader to investigate (Castellino, 2005; Lehman et al., 2015). CAD can either be presented simultaneously with the mammogram or be presented after a reader has initially searched the display to act as a decision aid (e.g. Hupse et al., 2013). CAD is regularly used in the USA and has been adopted (or considered for adoption) in other countries too (e.g. Guerriero et al., 2011; Houssami et al., 2009; Lehman et al., 2015; Sato et al., 2014). Within the USA, CAD is used in the majority of Medicare screening populations (Lehman et al., 2015) and its use has increased over the past two decades with advancements in AI and substantial financial investments in this technology (e.g. Keen et al., 2018; Henriksen et al., 2019; Elmore & Lee, 2022; Harvey et al., 2019). However, the benefits and costs of CAD have been debated with some studies declaring either little, no or inconclusive benefit (e.g. Azavedo et al., 2012; Bennett et al., 2006; Gilbert et al., 2008; Lehman et al., 2015), while others have suggested positive outcomes of introducing CAD (e.g. Samulski et al., 2010; Zheng et al., 2004). A range of methodologies have been used to investigate CAD including clinical observational studies, Randomised Clinical Trials (RCTs) and laboratory-based studies. Laboratory studies are a good way of investigating the effects of CAD, to complement clinical studies, as they allow the recruitment and participation of more readers than typically available for clinical observational studies, in a time efficient manner (RCTs often take years to run, at which point the CAD systems being tested may be obsolete).

Using laboratory-based studies, Kunar et al. (2017) evaluated the effectiveness of CAD and showed that along with its benefits, readers showed substantial over-reliance effects on the technology, leading to a high proportion of both miss errors and false alarms when the CAD technology was wrong. In their study, Kunar et al. (2017) trained naïve readers to find cancers in a mammogram search task (adapted for use with non-medical readers). The results showed that when the cancer was highlighted by the CAD cue, cancer detection was high, with very few missed cancers. However, when the CAD cue was inaccurate (either by failing to highlight the cancer or highlighting a non-cancerous area), readers missed a large proportion of cancers and showed greater miss errors compared to when a CAD system was never used (see also Drew et al., 2020). Furthermore, participants made more false alarms (saying that a cancer was present when there was not one) when a CAD cue was incorrectly presented on a target absent trial. In a clinical setting, although miss errors (where a cancer goes unnoticed) may ultimately be the more serious type of error, false alarms also have their own negative consequences. For example, false alarms lead to unwarranted anxiety for the women involved (Aro, 2000), a delay in the uptake of future mammogram scans (Kahn & Luce, 2003), and increased unnecessary financial healthcare costs (as women are incorrectly recalled for follow-up, often invasive tests)—all of which add burden to already over-stretched healthcare systems. Given that CAD accuracy is variable, and no CAD system is (of yet) 100% reliable, these over-reliance effects on CAD are of concern. Please note that human radiologists are also not 100% reliable in detecting cancers over a long period of time. However, as CAD systems are designed to help human readers and decrease detection errors, it is important to assess and minimise any extra concerns (such as over-reliance effects) that CAD may introduce to cancer detection.

Despite this, when CAD cues are correct, they are beneficial to the reader in terms of leading to a reduction in miss errors (Kunar et al., 2017; Kunar, 2022; Russell & Kunar, 2012; Drew et al., 2020). This is particularly important when we consider the prevalence of the targets that readers are searching for. Wolfe et al. (2005) have demonstrated the existence of a Low Prevalence (LP) effect whereby miss errors increase dramatically with a decrease in target prevalence rate (see also Wolfe et al., 2007; Rich et al., 2008; Kunar et al., 2010, 2021; Russell & Kunar, 2012; Van Wert et al., 2009; Mitroff & Biggs, 2014). This effect has been shown to be robust and immune to a large range of interventions that try and decrease the LP effect (Wolfe et al., 2007). Given that the prevalence of cancers in mammography screening is low (Gur et al., 2003), it is crucial to find ways to help readers detect cancers when they are present. One such way to reduce the LP Effect is with the use of CAD (at least when the algorithm is accurate). Given this benefit and the recent world-wide investment in Machine Learning and AI within health care, it would be prudent to investigate ways to improve human interaction with CAD to keep the benefits, while reducing any costs of user over-reliance in such systems.

In one such study, Kunar (2022) found that the way CAD was presented to the reader affected search, so that if CAD was presented *after* observers had examined the initial mammogram, fewer miss errors were found compared to when CAD was presented *simultaneously* with the mammogram (see also Drew et al., 2020 who looked at CAD presentation). This showed that the effectiveness of CAD was malleable, and its presentation could be adjusted for optimal human interaction. The current study investigates another way that human–CAD interaction might be affected by examining whether framing of the CAD’s effectiveness can reduce over-reliance on this technology.

Previous research has shown that the presence of advanced knowledge or training prior to a task can mitigate cognitive biases and has been proposed as a beneficial way to improve decision making in both applied settings and when technology is involved (Kassin et al., 2013; Sellier et al., 2019). Furthermore, there is a substantial body of literature that shows (1) the way information is framed can have large impacts on human behaviour (e.g. Thaler & Sunstein, 2008) and (2) additional instruction, information and expectations can affect how people search visual displays and make medical decisions (Cox et al., 2021; Madrid & Hout, 2019; Phelps et al., 2017). Therefore, we investigate whether framing information about either the benefits or costs of CAD will keep the advantages, while mitigating the over-reliance effects. In particular, if people are told that CAD can sometimes be inaccurate and lead to missed cancers, will they be less likely to show this over-reliance bias?

Of course, it is also important to establish that framing information about the costs of CAD will not undermine the benefits of CAD when it is accurate. However, we hypothesise that this will not be the case. To be effective, CAD prompts need to be salient to alert a reader to a possible target—and therefore, exogenous cues are often used. Previous research in the visual search literature suggests exogenous cues capture attention in a bottom-up fashion and, as such, are not easily ignored (in some instances it is impossible to ignore these types of cues, e.g. Remington et al., 1992; Theeuwes, 1994). More recently, it has been found that bottom-up signals of distracting information can be attentionally suppressed by top-down control (e.g. Sawaki & Luck, 2010; Gaspelin et al., 2017; Wang & Theeuwes, 2018). However, when salient signals are not suppressed, they will elicit increased activation in a Priority Map, (e.g. Wolfe, 2021; Luck et al., 2021). By this account, CAD cues will benefit search if they highlight the target accurately. Conversely, if the CAD cue is deemed irrelevant, then attention will move to the next highest peak on the Priority Map and search will continue until the target is found or a quitting threshold has been reached to stop search (Wolfe, 2021). This process of ‘search beyond the cue’ is important to ensure that that attention is not ‘captured’ by the CAD cue for the whole search task once it is deemed irrelevant. This implies that any cue should be capable of releasing attention after the initial capture (i.e. designing and presenting a cue which continually drew attention to itself throughout the reading period would not be wise). We hypothesise that any CAD prompt will initially generate a large priority signal that will draw attention and cause its immediate area to be searched. Therefore, miss errors should not be affected when the CAD cue predicts a target. However, if people have prior knowledge that CAD cues are fallible, they may be more likely to search the display more thoroughly than if they are not told of its inaccuracies.

We present three experiments that examine how framing the costs or benefits of CAD affects target detection. In Experiment 1, people were given advanced knowledge that CAD can be useful for finding the target (i.e. framing the benefits of CAD) or that CAD can cause people to miss the target if it is inaccurate (i.e. framing the costs of CAD). Experiment 2 was similar to Experiment 1 but gave a stronger warning of the benefits/costs and explicitly instructed the reader to either use or ignore CAD. The results showed that whereas a mild warning of the costs/benefits of CAD did little to mitigate the over-reliance effect, a stronger warning and instruction set resulted in fewer false alarms and a reduction in miss errors for Incorrect CAD cues, whilst retaining the benefit of CAD when it was correct. Experiment 3 replicated Experiment 2, but while Experiments 1 and 2 used a high target prevalence (a target was present on 50% of trials), Experiment 3 looked at the effect of a strong warning when the target had a lower prevalence rate (the target was present 10% of the time). Horowitz (2017) highlights the importance of testing CAD under LP rates, given that search mechanisms differ across prevalence rates.^1^ Again, to preview the results, giving people a strong warning and instruction set of CAD costs led to fewer false alarms at LP (but not a reduction in miss errors), while retaining the benefit when CAD was correct. Collectively, the results show that despite CAD prompts having a strong attentional capture effect, leading to an over-reliance effect across experiments, framing about the costs of CAD reduced this over-reliance, while retaining the benefits when the CAD technology was correct.

## Experiment 1

### Method

#### Transparency and openness

The data can be found on the Open Science Framework (https://osf.io/m832p/). All data were compiled in Microsoft® Excel® for Microsoft 365 MSO (Version 2112 Build 16.0.14729.20254) and imported into SPSS (Version 27, Release 27 0.1.0) and JASP (Version 0.16; JASP Team, 2021) for statistical analysis. The experimental programs were written in BlitzMax (Version 1.48 Sibly, 2004). The study design, hypotheses and analytic plan were not pre-registered. All manipulations, data exclusions and measures are reported.

##### Participants

Forty participants (mean age = 24.1 years) took part in Experiment 1. Participant numbers were pre-determined prior to study commencement and were based on participant numbers used in previous research (e.g. Drew et al., 2012; Kunar et al., 2017; Wolfe et al., 2007). A power analysis calculated using G*Power (F-tests, effect size = 0.25, alpha = 0.05, see Faul et al., 2007) showed that the minimum number of participants needed to achieve a power of 0.8, for each condition was 14. Therefore, testing 20 participants per condition should provide sufficient power to detect any significant effects. In all experiments, participants were recruited from the University of Warwick participant pool, had no prior training in reading mammograms and were paid for their time. Ethical approval for all studies was granted by the Humanities and Social Sciences Research Ethics Committee at the University of Warwick.

##### Stimuli and procedure

The mammogram images were taken from the selection of ‘normal’ mammograms (those not containing a cancer) of the Digital Database for Screening Mammography (DDSM) database (Heath et al., 1998, 2001). All images were selected at random. Images were presented in the centre of the display and subtended approximately 11 degrees by 19 degrees at a viewing distance of 57 cm (although the individual size of each image varied because they were real mammograms). For target present trials, four cancers and four benign masses were selected at random from cancer cases and benign cases on the DDSM. In radiology, a mass can take on multiple different forms and requires years of training to detect. As the participants in these experiments were medically untrained, we chose to use four examples of each mass, throughout the experiment (i.e. in training, practice and experimental trials) to train participants on and act as targets. This ensured that participants knew what to search for and follows the methodology of other research which used examples of discrete and detectable stimuli as targets (e.g. Drew et al., 2012, 2020; Kunar, 2022; Kunar et al., 2017, 2021; Rich et al., 2008; Wolfe et al., 2005, 2007 etc.). These masses were then transposed onto mammograms that previously contained no cancer using imaging editing software so that each image contained one mass (either cancerous or benign, each mass appeared equally often throughout the experiment). The mass could appear on any area of the breast tissue, chosen at random (mimicking conditions in a clinical setting), provided that it was clearly distinguishable once fixated (see also Kunar et al., 2017; Kunar et al., 2021; Kunar, 2022). The CAD cues consisted of a red outline box that subtended 1.1 degrees by 1.1 degrees at a viewing distance of 57 cm. All mammogram images were created prior to the experiment.

**Fig. 1:**
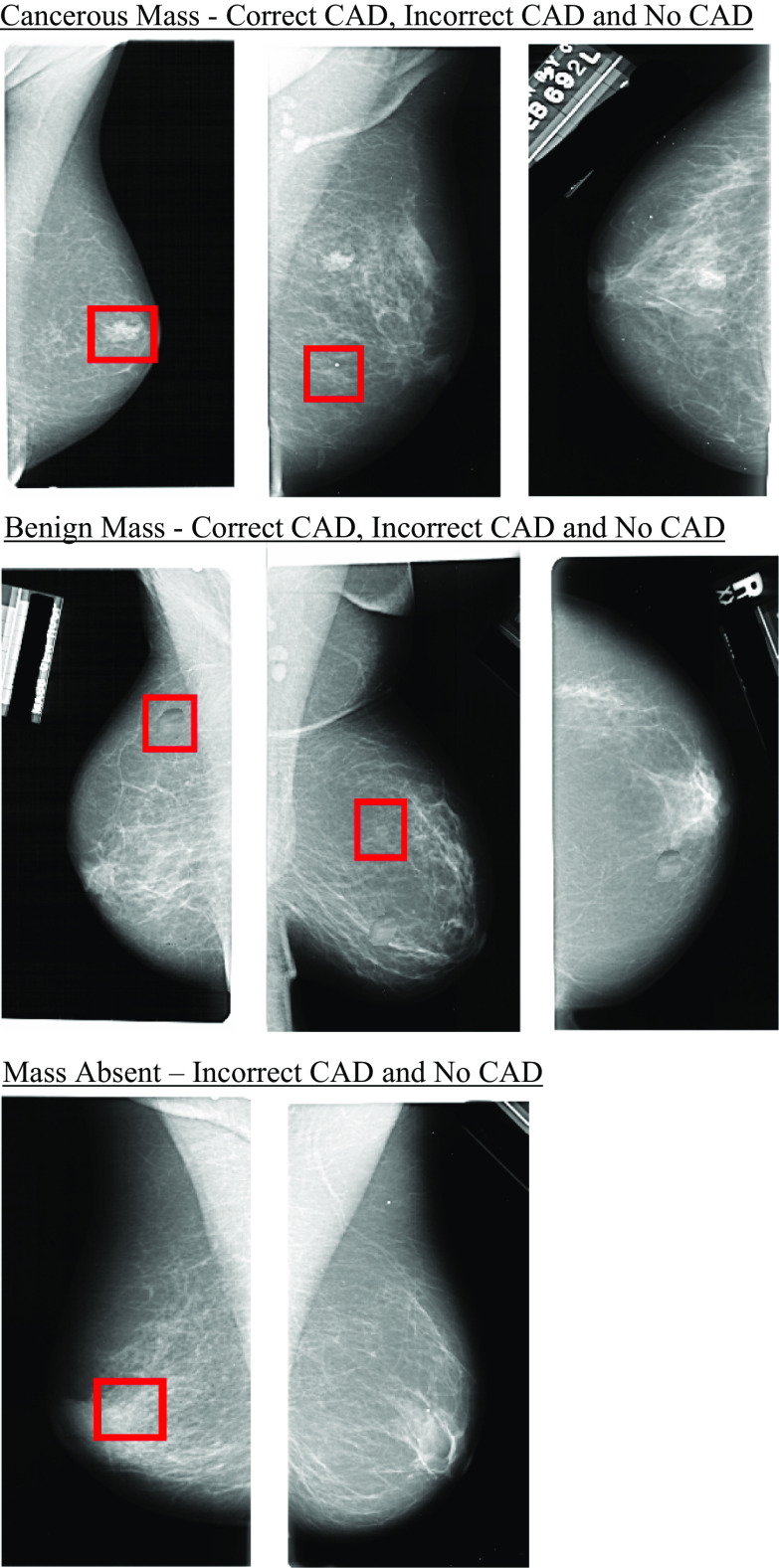
Examples of mammogram displays with Correct CAD, Incorrect CAD and No CAD for cancerous, benign and no mass (target absent) conditions

In each condition, there were 200 target absent trials and 200 target present trials. For the target absent trials, 150 trials (75%) were presented without any CAD cues (No CAD). The other 50 trials (25%) of target absent trials contained a CAD cue placed on a random area of the mammogram (representing an incorrect CAD cue, see also Russell & Kunar, 2012; Kunar et al., 2017 for similar methodology). For target present trials, 100 trials contained a benign mass and 100 trials contained a cancer. Within the benign mass trials, 60 trials showed a CAD cue that correctly highlighted the mass (Correct CAD), 20 trials showed a mass that fell outside of the CAD cue, with the CAD placed on another random area within the breast tissue (Incorrect CAD), and 20 trials contained a mass but did not show any CAD cue (No CAD). The same proportion of Correct, Incorrect and No CAD trials were used for mammograms that contained a cancer. CAD accuracy has been shown to vary from 57% (Soo et al., 2005) to 85% (Obenauer et al., 2006, see also Henriksen et al., 2019 who report a CAD accuracy of between 65 and 77%) depending on the type of cancer and suspiciousness of the lesion. Therefore, we chose an accuracy rate of 60% for target present trials to fit within this range (see also methodology of Russell & Kunar, 2012; Kunar et al., 2017; Kunar, 2022). Participants viewed all 400 mammogram images presented in a random order (see Fig. 1 for an example image).

Before taking part in the experiment proper, participants were given a training session to familiarise themselves with mammogram images and the cancers and benign masses. In this training session, participants were first shown images of both the cancerous and benign masses on their own. The experimenter gave participants information of what to look for (e.g. the cancers have a more spiculated appearance than benign masses, to help participants we described cancer masses as having a more textured and ‘striped’ or ‘spiky’ appearance than benign masses). They were then shown 24 different mammograms, each containing a mass. The first 12 contained a benign mass; the next 12 contained a cancer. Participants were asked to point to the mass in each mammogram while the experimenter was in the same room (the experimenter would provide feedback if needed). Once participants completed this cancer identification task and both the participant and experimenter were confident that the participant could identify a mass, they proceeded to take a ‘test-identification’ block, where they were shown 24 mammograms, each containing a mass and asked to identify the mass as either a cancer or benign mass (by pressing keys c or b, respectively, on a standard computer keyboard). The experimenter remained in the room during this time to make sure the participant could categorise the masses correctly. If a participant had difficulty identifying the mass, they could be shown more examples and could repeat the training condition until both the participant and experimenter were confident that they were able to identify the mass. However, in practice, all the participants learned to identify the mass within the first session and none were asked to repeat it. Once this training phase was complete, participants could then begin the full experiment. None of the mammograms used in the training phase or the practice were repeated in the main experiment.

There were two between-subjects experimental conditions: a *Frame Benefits* condition and a *Frame Costs* condition. Half the participants completed the Frame Benefits condition; half the participants completed the Frame Costs condition. At the start of each condition, participants were shown a set of instructions asking them to search for a mass that could appear in the image. Participants were then shown an instruction page outlining either the benefits or costs of CAD. The instructions read: “We will be examining how Computer-Aided Detection (CAD) cues help people find cancers. The CAD cues are red boxes that sometimes highlight where a cancer is.” For the Frame Benefits Condition, participants then saw a sentence stating: “The CAD cues have been found to greatly help people find a cancer when it is present”. For the Frame Costs Condition, participants were shown a sentence stating: “However, the CAD cues are likely to cause you to miss a cancer if it is incorrectly used”. Both critical sentences were highlighted to participants in their respective condition using bold and red ink. On a final instruction page, participants were asked to respond as accurately as possible and reminded of the message framing CAD in terms of either its benefits or costs.

Within each trial, participants were first shown a black screen for 500 ms. They were then presented with one of the mammogram images. CAD cues were automatically presented at the same time as the mammogram. Participants were asked to respond whether they thought a mass was present or absent by pressing either the ‘m’ or the ‘z’ key, respectively, which then removed the display. If participants responded that a mass was present, they were then asked whether it was cancerous or benign and responded by pressing either ‘c’ or ‘b,’ respectively. The experiment then moved onto the next trial. If participants responded that a mass was absent, the experiment moved onto the next trial. If no response was made within 10 s, the trial ‘timed-out’ and the next trial started automatically. Following a response or ‘time-out’, a black screen was again displayed before the next trial. In both conditions, Reaction Times (RTs) and error rates were recorded. RTs indicating a timeout and those less than 200 ms were considered outliers and removed from data analysis. If participants realised they had pressed the wrong button (e.g. pressed that the target was absent rather than present, due to a motor error), they were able to correct it on the following trial, by pressing the ‘Escape’ key during any time of the next trial (see Fleck & Mitroff, 2007; Van Wert et al., 2009; Kunar et al., 2010; Kunar et al., 2017; Kunar et al., 2021; Russell & Kunar, 2012; Rich et al., 2008, for similar methodologies). This did not affect the presentation of the next trial, but was recorded in the data file, and allowed participants to correct any motor errors. It also enabled self-corrected responses to be subsequently calculated. Participants then continued with the current trial, responding with an ‘m’ or ‘z’ key if the target was present or absent, respectively. No feedback was given after any response, or correction, was made. As the results of interest are from cognitive rather than motor response errors (i.e. those that can be corrected in the field), the analyses were conducted using the self-corrected data (see also Kunar et al., 2017, 2020; Kunar, 2022). For both conditions, participants completed a short practice block before they started the experimental block of trials. Like the experimental block, the practice block included displays that contained a mass (either a cancer or benign) and those that did not. This ensured that participants were shown examples of all types of mammograms (i.e. both target present and target absent) prior to completing the experimental trials.

Across experiments, there are a number of statistical analyses that could be conducted; however, we limited our analyses to those addressing the question at hand. Where planned t-tests are reported, we also include Bayesian analyses, as supportive evidence (Wagenmakers et al., 2018a). We only include Bayesian analysis for t-tests rather than ANOVAs as the latter is still an ongoing topic of research (Wagenmakers et al., 2018b). For our Bayesian analyses, we adopt the recommendations of Jeffreys (1961) in which a BF_10_ of 1–3 provides *anecdotal* evidence for the alternative, a BF_10_ of 3–10 provides *substantial* evidence for the alternative, a BF_10_ of 10–30 provides *strong* evidence for the alternative, a BF_10_ of 30–100 provides *very strong* evidence for the alternative and a BF_10_ of greater than 100 provides *decisive* evidence for the alternative. The inverse of these numbers (BF_01_) provides evidence in support the null hypothesis (Jarosz & Wiley, 2014).

### Results

**Fig. 2:**
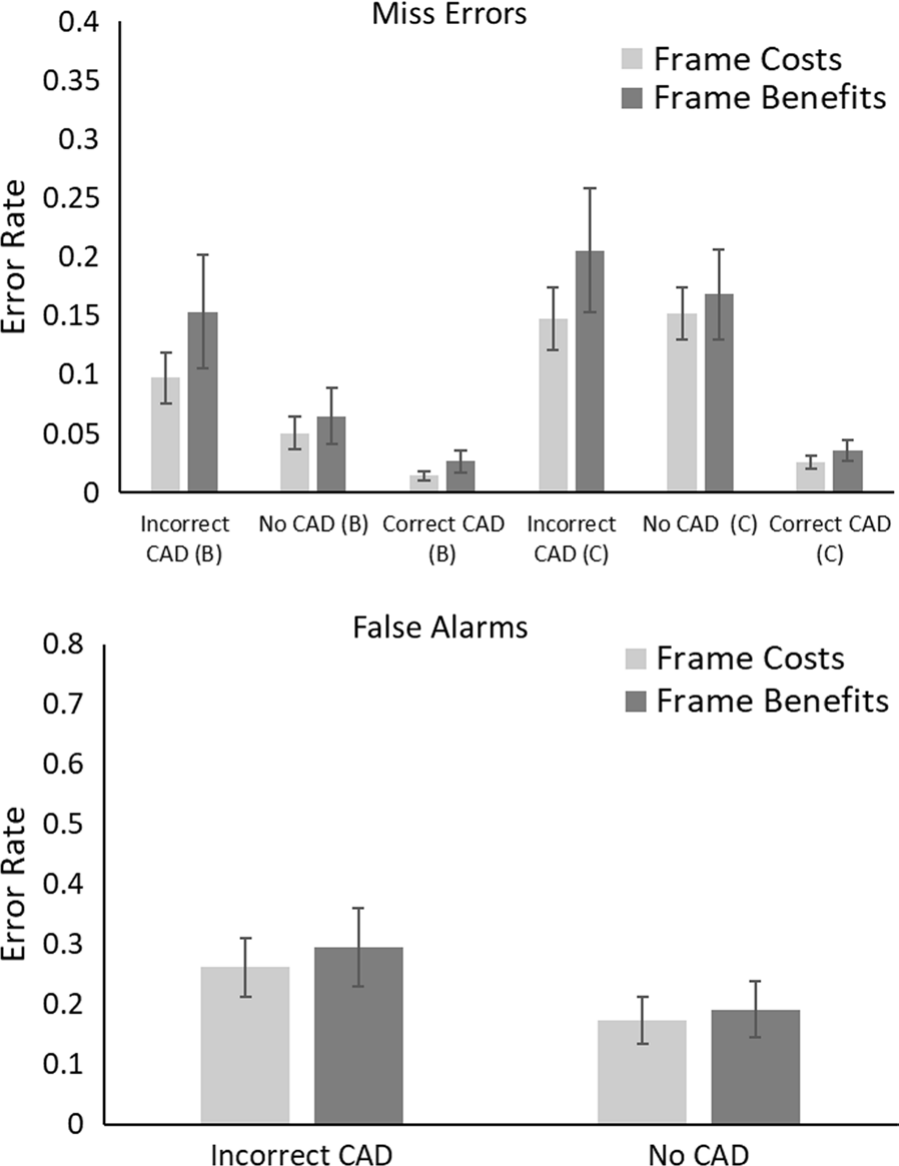
Proportion of miss errors and false alarms in Experiment 1. Trials containing a benign mass as a target are denoted with (B); trials containing a cancer as a target are denoted with (C). Error bars represent the standard error

The outlier procedure removed 1.49% of all data. Error rates for all conditions are presented in Fig. 2.

#### Miss errors

A 2 × 3 × 2 mixed ANOVA was conducted on miss errors (calculated from target present trials only) with within-participant factors of Mass (Cancer vs. Benign) and CAD (Correct CAD, Incorrect CAD and No CAD) and between participant factor of Framing (Benefits vs. Costs). This revealed a significant main effect of Mass, *F*(1, 38) = 40.36, *p* < 0.001, *η*_*p*_^2^ = 0.52, in which participants missed more cancers than benign masses. There was a significant main effect of CAD, *F*(2, 76) = 18.23, *p* < 0.001, *η*_*p*_^2^ = 0.32, in which there were fewest misses in the Correct CAD and most misses in the Incorrect CAD conditions. There was no significant effect of Framing, *F*(1, 38) = 1.0, *p* = 0.32, *η*_*p*_^2^ = 0.03. There was a significant Mass × CAD interaction, *F*(2, 76) = 21.73, *p* < 0.001, *η*_*p*_^2^ = 0.36, in which the difference in miss errors across CAD conditions was larger when the mass was a cancer compared to when it was benign. None of the other interactions, including those with Framing as a factor, were significant (all Fs < 1, ps > 0.49).

#### False alarms

A 2 × 2 mixed ANOVA on false alarms (calculated from target absent trials only)^2^ with within-participant factors of CAD (No CAD vs. Incorrect CAD) and between participant factor of Framing (Benefits vs. Costs) revealed a significant main effect of CAD, *F*(1, 38) = 20.05, *p* < 0.001, *η*_*p*_^2^ = 0.35; there were more false alarms with Incorrect CAD compared to No CAD. There was no main effect of Framing, *F*(1, 38) = 0.14, *p* = 0.71, *η*_*p*_^2^ = 0.004. The CAD × Framing Interaction was not significant, *F*(1, 38) = 0.14, *p* = 0.71, *η*_*p*_^2^ = 0.004.

#### Signal detection theory

**Fig. 3:**
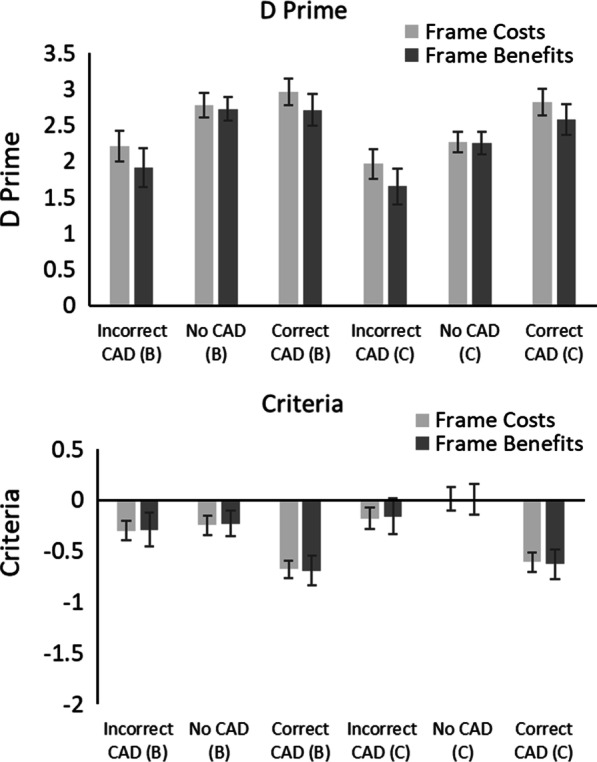
D’ and c values in Experiment 1. Trials containing a benign mass as a target are denoted with (B); trials containing a cancer as a target are denoted with (C). Error bars represent the standard error

Signal Detection Theory (SDT, Green & Swets, 1967; Macmillan & Creelman, 2005) was used to calculate how CAD and prior knowledge influenced *d’* (a change in sensitivity) and *c* (a change in criterion).^3^ Figure 3 shows the *d’* and *c* values.

#### Sensitivity (*d*’)

A 2 × 3 × 2 mixed ANOVA on *d’* with within-participant factors of Mass (Cancer vs. Benign) and CAD (Correct CAD, Incorrect CAD and No CAD) and between participant factor of Framing (Benefits vs. Costs) revealed a significant main effect of Mass, *F*(1, 38) = 34.78, *p* < 0.001, *η*_*p*_^2^ = 0.48, in which *d’* was greater for benign masses than cancers. There was a significant main effect of CAD, *F*(2, 76) = 30.94, *p* < 0.001, *η*_*p*_^2^ = 0.45, in which *d’* was greatest for Correct CAD, followed by No CAD and then Incorrect CAD. There was no significant effect of Framing, *F*(1, 38) = 0.62, *p* = 0.44, *η*_*p*_^2^ = 0.02. There was a significant Mass × CAD interaction, *F*(2, 76) = 12.36, *p* < 0.001, *η*_*p*_^2^ = 0.25, in which *d’* for cancers was lower than that for benign masses in the No CAD and Incorrect CAD conditions but not in the Correct CAD condition. No other interactions were significant (all Fs < 1, ps > 0.43).

#### Criteria, (*c*)

A 2 × 3 × 2 mixed ANOVA on *c* with within-participant factors of Mass (Cancer vs. Benign) and CAD (Correct CAD, Incorrect CAD and No CAD) and between participant factor of Framing (Benefits vs. Costs) revealed a significant main effect of Mass, *F*(1, 38) = 34.77, *p* < 0.001, *η*_*p*_^2^ = 0.48, in which participants were more willing to respond that a benign mass was present compared to a cancer. There was a significant main effect of CAD, *F*(2, 76) = 52.69, *p* < 0.001, *η*_*p*_^2^ = 0.58, in which participants were more willing to respond that a mass was present with Correct CAD compared to Incorrect and No CAD conditions. There was no significant effect of Framing, *F*(1, 38) = 0.00, *p* = 0.99, *η*_*p*_^2^ = 0.00. There was a significant Mass × CAD interaction, *F*(2, 76) = 12.36, *p* < 0.001, *η*_*p*_^2^ = 0.25, in which participants were more willing to respond to benign masses than cancers in the No CAD and Incorrect CAD conditions but not in the Correct CAD condition. No other interactions were significant (all Fs < 1, ps > 0.92).

#### Mass identification errors

**Fig. 4:**
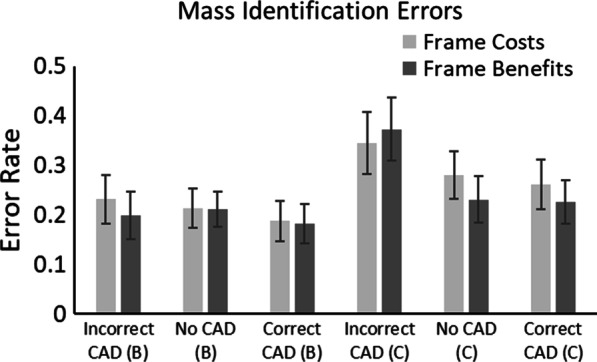
Proportion of mass identification errors in Experiment 1. Trials containing a benign mass as a target are denoted with (B), trials containing a cancer as a target are denoted with (C). Error bars represent the standard error

The effect of CAD and Framing on mass identification errors was also examined (Fig. 4). A 2 × 3 × 2 mixed ANOVA on mass identification errors with within-participant factors of Mass (Cancer vs. Benign) and CAD (Correct CAD, Incorrect CAD and No CAD) and between participant factor of Framing (Benefits vs. Costs) revealed a significant main effect of Mass, *F*(1, 38) = 4.68, *p* = 0.037, *η*_*p*_^2^ = 0.11; participants made more errors in identifying cancers than benign masses. There was also a significant main effect of CAD, *F*(2, 76) = 10.62, *p* < 0.001, *η*_*p*_^2^ = 0.22; Mass Identification Errors were highest with Incorrect CAD and lowest with Correct CAD. There was no main effect of Framing, *F*(1, 38) = 0.12, *p* = 0.74, *η*_*p*_^2^ = 0.003. The Mass × CAD interaction was significant, *F*(2, 76) = 4.80, *p* = 0.01, *η*_*p*_^2^ = 0.11, in which Incorrect CAD led to an increase in cancers being misidentified, but not benign masses. None of the other interactions were significant, (all Fs < 1.4, ps > 0.26).

### Discussion

Experiment 1 examined whether framing the benefits or costs of CAD would mitigate their over-reliance on CAD technology, by reducing the miss errors and false alarms when CAD cues were incorrect. The results showed that there was no difference in miss errors or false alarms between participants who were told the benefits of using CAD and those that were told of its costs.

Replicating previous results (Kunar, 2022; Kunar et al., 2017), in all conditions, there was an over-reliance effect of CAD. There were fewer miss errors when CAD was correct, compared to when CAD was incorrect or no CAD was presented. Having an Incorrect CAD cue also led to a greater number of false alarms. However, framing the costs/benefits of CAD cues did statistically nothing to alleviate over-reliance on this technology.

Results from the SDT analysis also showed that CAD affected both sensitivity (as measured by *d’*) and participants’ response criteria (as measured by *c*). Correct CAD cues led to both an increase in sensitivity and a shift in response threshold so that participants were more willing to respond that a target was present. The same pattern of results was found for both framing conditions regardless of whether participants were told about either the benefits or costs of CAD.

Across all conditions, there was a difference in how participants responded to cancers versus benign masses. Participants made fewer miss errors, showed greater sensitivity and were more willing to say a mass was present when the target was benign in comparison to when it was a cancer. This effect is similar to that found in previous work which also used simulated mammograms (Kunar et al., 2017) and may be a result of the mass exemplars used. For example, the benign masses were less spiculated than their cancerous counterparts, resulting in a smoother texture. This difference may have led them to be more easily segmented from the background leading them to greater detection of benign masses (e.g. Julesz, 1981, see also Kunar et al., 2017).

Examining mass identification errors, cancers were misidentified more often than benign masses. There was also an effect of CAD on mass identification—participants were more likely to misidentify a mass if the CAD cue was Incorrect. This was particularly the case for cancerous masses—where participants misidentified the mass as being benign if a CAD cue was present but highlighting an area without an anomaly. In this experiment, not only did an Incorrect CAD cue lead to an increase in miss errors—it had a detrimental effect on identification errors so a mass falling outside a CAD cue might be more likely to be perceived as ‘harmless’ or benign. These data suggest that the over-reliance participants showed about CAD may also affect identification errors, alongside search errors (see Kunar et al., 2017 for similar evidence). We discuss this further in the General Discussion.

Experiment 1 showed that participant’s over-reliance on CAD was not changed by framing of the CAD benefits or costs. Experiment 2 investigated whether people showed the same level of over-reliance on CAD when the warnings about CAD costs were made stronger, and they were explicitly told not to use them.

## Experiment 2

### Method

#### Participants

Forty participants (Mean age = 18.5 years) took part in Experiment 2. Half the participants took part in the Frame Benefits Condition, and half the participants took part in the Frame Costs condition.

#### Stimuli and procedure

The stimuli and procedure were the same as Experiment 1, apart from the following. As there was no effect of mass type on knowledge, on target present trials, we chose to only present mammograms containing a cancer in Experiment 2 and the remaining experiments (rather than cancers and benign masses). Participants were still given a training phase to familiarise themselves with mammogram and cancer images (however, they were not given examples of benign images or trained to distinguish between a cancer and benign mass in the training phase). This meant that each experiment condition contained 200 trials—100 target present and 100 target absent. For the target present trials, 60 contained the cancer in the CAD (Correct CAD), 20 contained the cancer outside the CAD box (Incorrect CAD) and 20 contained no CAD cues (No CAD). For the target absent trials, 25 contained a CAD cue (Incorrect CAD) and 75 contained no CAD (No CAD).

Similar to Experiment 1, participants took part in one of two conditions: a Frame Benefits condition or a Frame Costs condition. However, the severity of the warning in the message was increased. In the Frame Benefits condition, participants were given the explicit instructions before the experiment stating that: “The CAD cues have been found to greatly help people find a cancer when it is present. They have been used to accurately predict where the cancer is. Please use the CAD cues to help find the target”. In the Frame Costs condition, participants were given the explicit instructions before the experiment that: “It has been shown that people can incorrectly rely on the CAD cues and not search the mammogram properly. Often the CAD cues are INCORRECT. Please IGNORE the CAD cues and DO NOT use them”. On a final instruction page, participants were asked to respond as accurately as possible and reminded of the message framing CAD in terms of either its benefits or costs.

### Results

**Fig. 5:**
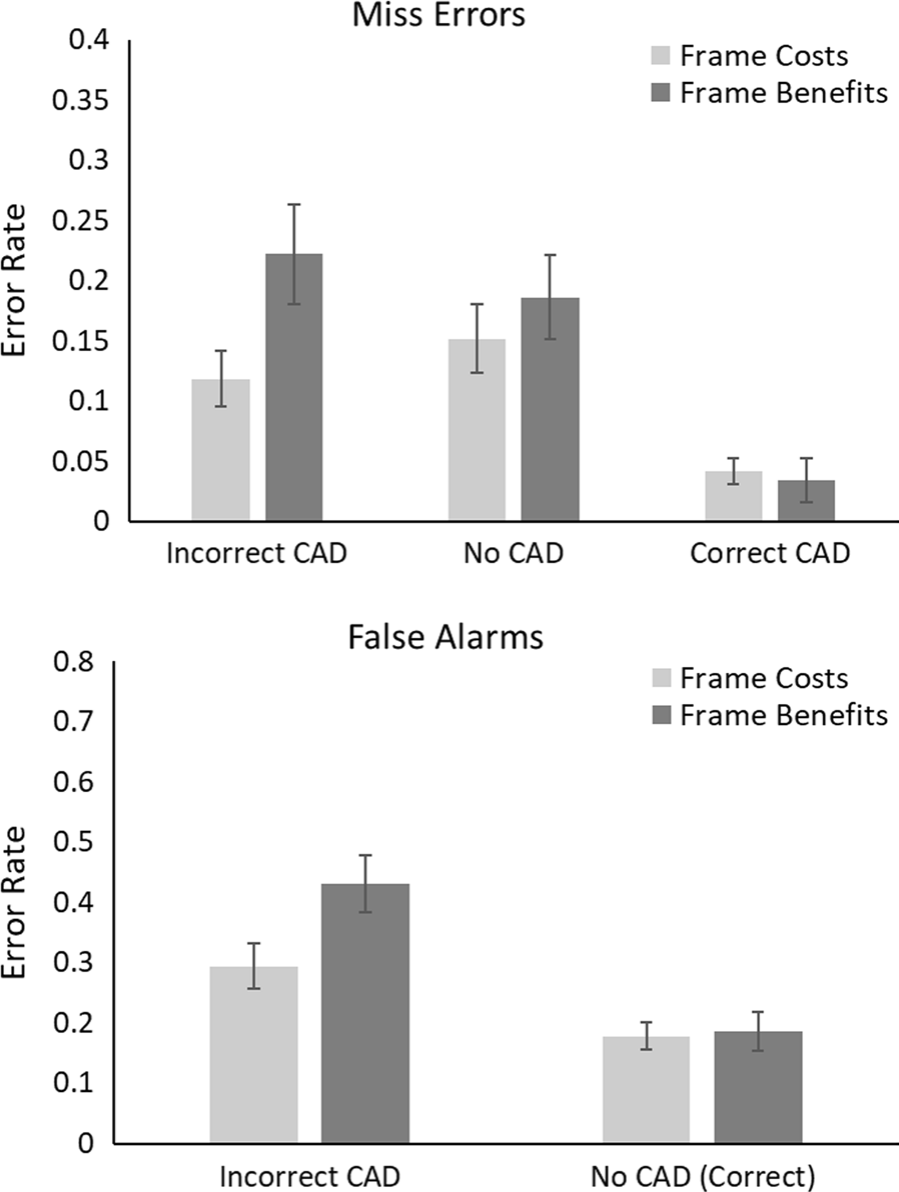
Proportion of miss errors and false alarms in Experiment 2. Error bars represent the standard error

The outlier procedure removed 1.09% of all data. Error rates for all conditions are presented in Fig. 5.

#### Miss errors

A 3 × 2 mixed ANOVA on miss errors with within-participant factors of CAD (Correct CAD, Incorrect CAD and No CAD) and between participant factor of Framing (Benefits vs. Costs) revealed a significant main effect of CAD, *F*(2, 76) = 30.87, *p* < 0.001, *η*_*p*_^2^ = 0.45, in which there were fewest miss errors in the Correct CAD compared to the No CAD and Incorrect CAD conditions. There was no significant effect of Framing, *F*(1, 38) = 1.77, *p* = 0.19, *η*_*p*_^2^ = 0.04. However, there was a significant CAD × Framing interaction, *F*(2, 76) = 4.21, *p* = 0.018, *η*_*p*_^2^ = 0.10. Given the CAD × Framing interaction was significant, planned t-tests were used to break-down this interaction. For Incorrect CAD participants made fewer miss errors in the Frame Costs compared to the Frame Benefits Condition, t(38) = 2.19, *p* = 0.04, d = 0.69, with anecdotal evidence in support of the alternative, BF_10_ = 1.94. There was no difference in miss errors for Correct CAD, t(38) = 0.34, *p* = 0.73, d = 0.11, with substantial evidence in support of the null, BF_10_ = 0.32, or No CAD conditions, t(38) = 0.77, 0 = 0.45, d = 0.24, with anecdotal evidence in support of the null, BF_10_ = 0.39.

#### False alarms

A 2 × 2 mixed ANOVA on false alarms with within-participant factors of CAD (No CAD vs. Incorrect CAD) and between participant factor of Framing (Benefits vs. Costs) revealed a significant main effect of CAD, *F*(1, 38) = 68.06, *p* < 0.001, *η*_*p*_^2^ = 0.64; there were more false alarms with Incorrect CAD compared to No CAD. There was no main effect of Framing, *F*(1, 38) = 2.52, *p* = 0.12, *η*_*p*_^2^ = 0.06. However, the CAD × Framing Interaction was significant, *F*(1, 38) = 8.73, *p* = 0.005, *η*_*p*_^2^ = 0.19. Given the CAD ×  Framing interaction was significant, planned t-tests were used to break-down this interaction. For Incorrect CAD, participants made fewer false alarms in the Frame Costs compared to the Frame Benefits Condition, t(38) = 2.29, *p* = 0.03, d = 0.72, with anecdotal evidence in support of the alternative BF_10_ = 2.29. As would be expected, there was no difference in false alarms across Framing conditions when there was No CAD, t(38) = 0.21, *p* = 0.84, d = 0.07, with substantial evidence in support of the null, BF_10_ = 0.31.

#### Signal detection theory

**Fig. 6:**
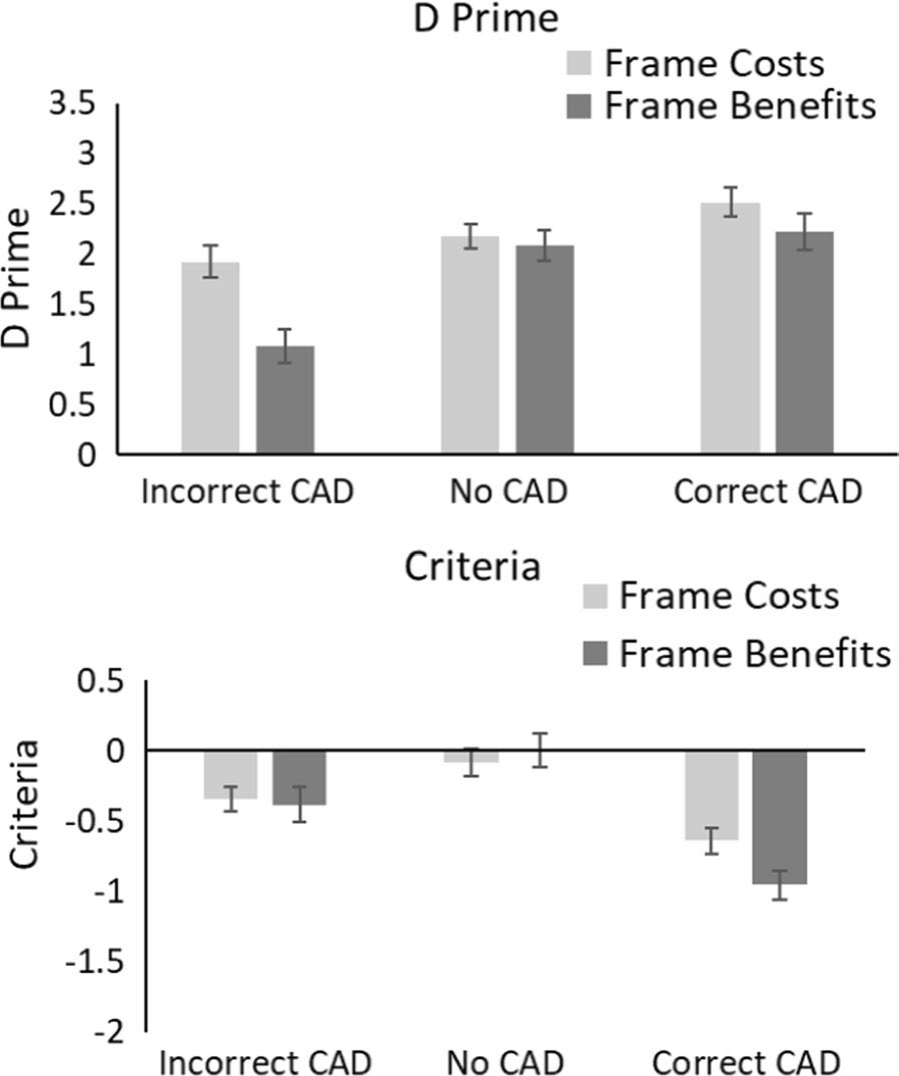
D’ and c values in Experiment 2. Error bars represent the standard error

Figure 6 shows the *d’* and *c* values.

#### Sensitivity (*d*’)

A 3 × 2 mixed ANOVA on *d’* with within-participant factors of CAD (Correct CAD, Incorrect CAD and No CAD) and between participant factor of Framing (Benefits vs. Costs) revealed a significant main effect of CAD, *F*(2, 76) = 46.09, *p* < 0.001, *η*_*p*_^2^ = 0.55, in which *d’* was greatest for Correct CAD, followed by No CAD and then Incorrect CAD. There was a significant effect of Framing, *F*(1, 38) = 4.46, *p* = 0.04, *η*_*p*_^2^ = 0.11, in which *d’* was greatest in the Frame Costs condition compared to the Frame Benefits condition. There was also a significant CAD × Framing interaction, *F*(2, 76) = 8.60, *p* < 0.001, *η*_*p*_^2^ = 0.18. Given the CAD × Framing interaction was significant, planned t-tests were used to break-down this interaction. For Incorrect CAD, *d’* was greater in the Frame Costs compared to the Frame Benefits Condition, t(38) = 3.63, *p* < 0.001, d = 1.15, with very strong evidence in support of the alternative, BF_10_ = 36.09. There was no difference in *d’* across Framing conditions when CAD was correct, t(38) = 1.28, *p* = 0.21, d = 0.41, with anecdotal evidence in support of the null, BF_10_ = 0.59, or when there was No CAD, t(38) = 0.44, *p* = 0.66, d = 0.14, with anecdotal evidence in support of the null, BF_10_ = 0.33.

#### Criteria, (*c*)

A 3 × 2 mixed ANOVA on *c* with within-participant factors of CAD (Correct CAD, Incorrect CAD and No CAD) and between participant factor of Framing (Benefits vs. Costs) revealed a significant main effect of CAD, *F*(2, 76) = 107.87, *p* < 0.001, *η*_*p*_^2^ = 0.74, in which participants were more willing to respond that a mass was present with Correct CAD, followed by Incorrect and then No CAD. There was no significant effect of Framing, *F*(1, 38) = 0.41, *p* = 0.53, *η*_*p*_^2^ = 0.01. There was a significant CAD × Framing interaction, *F*(2, 76) = 7.97, *p* < 0.001, *η*_*p*_^2^ = 0.17. Given the CAD × Framing interaction was significant, planned t-tests were used to break-down this interaction. There was no difference in *c,* across Framing conditions for Incorrect CAD, t(38) = 0.27, *p* = 0.79, d = 0.09, with substantial evidence in support of the null, BF_10_ = 0.32, or when there was No CAD, t(38) = 0.58, *p* = 0.56, d = 0.18, with anecdotal evidence in support of the null, BF_10_ = 0.35. There was a difference in *c* across Framing conditions when CAD was correct, t(38) = 2.25, *p* = 0.03, d = 0.71, with anecdotal evidence in support of the alternative, BF_10_ = 2.17. Participants were more willing to state that a cancer was present in the Frame Benefits compared to the Frame Costs condition when the CAD cue was correct.

### Discussion

Experiment 2 showed that a stronger and more explicit instruction set, telling participants to either use or not use CAD, led to a change in participants’ over-reliance on CAD. Participants who were explicitly warned and given instructions in relation to the costs of CAD made fewer false alarms and miss errors when the CAD cue was incorrect. Importantly, in this experiment, such instruction did not lead to a difference in miss errors when the CAD cue was correct. Thus, giving people a strong warning and instruction set regarding the fallibility of CAD preserved its benefits but mitigated its costs.

Examining the SDT data, we can see that the difference in response behaviour for Incorrect CAD cues seems to be driven by *d’* rather than *c*. Framing the fallibility of CAD leads to an improvement in sensitivity and ability to detect the signal (i.e. cancer) from noise (i.e. areas not containing a cancer) when Incorrect CAD cues are present.

Please note that despite the improvement in sensitivity and reduction in errors when framing CAD’s costs, there was still an effect of CAD on search performance. People were still influenced by the presence of a CAD cue even when warned not to use it, as shown by the remaining presence of a (although somewhat attenuated) over-reliance effect. It seems that the presence of a CAD cue, even with warning not to use it, cannot help but affect mammogram reading. One potential reason for this could be that in these experiments, participants had a time limit of 10 s in which to respond before the display was removed. This is different from a clinical setting in which radiologists often spend longer searching individual mammograms. If participants felt pressurised into responding quickly, then they may be more likely to rely on the CAD recommendation. To examine this possibility, we looked at RTs for each framing condition. However, for both conditions participants responded well within the time limit (with an average of 1735 ms and 1599 ms, and a standard deviation of 954 ms and 715 ms, for the Frame Benefit and Frame Cost conditions, respectively) and therefore had ample time to examine each mammogram, before the display timed out. Thus, the over-reliance effect observed in this experiment does not seem to be driven by participants feeling a time pressure to respond.

Experiment 2 showed that framing the costs of CAD led to a reduction in the over-reliance effect. Please note that this Experiment had a target prevalence rate of 50%. Previous research has shown that search performance can differ between conditions in which a target appears frequently compared with when in occurs infrequently (high vs. low prevalence conditions). This finding led Horowitz (2017) to emphasise the importance of testing CAD under LP conditions. Accordingly, in Experiment 3, we replicated Experiment 2 under conditions where the target has a lower prevalence.

## Experiment 3

### Method

#### Participants

Forty participants (Mean age = 20.4 years) took part in Experiment 3. Half the participants completed the Frame Benefits Condition and half the Frame Costs condition.

#### Stimuli and procedure

The stimuli and procedure were the same as Experiment 2, apart from the prevalence of the target reduced from 50 to 10% and the time-out period for each trial being 15 s. In each condition, there were 1000 trials: 100 target present and 900 target absent. For the target present trials, 60 contained the cancer in the CAD (Correct CAD), 20 contained the cancer outside the CAD box (Incorrect CAD) and 20 contained no CAD cues (No CAD). For the target absent trials, 225 (25%) contained a CAD cue (Incorrect CAD) and 675 (75%) contained no CAD (Correct CAD). In each condition, participants were given the same framing instructions in relation to CAD as in Experiment 2 and were given breaks every 200 trials.

### Results

**Fig. 7:**
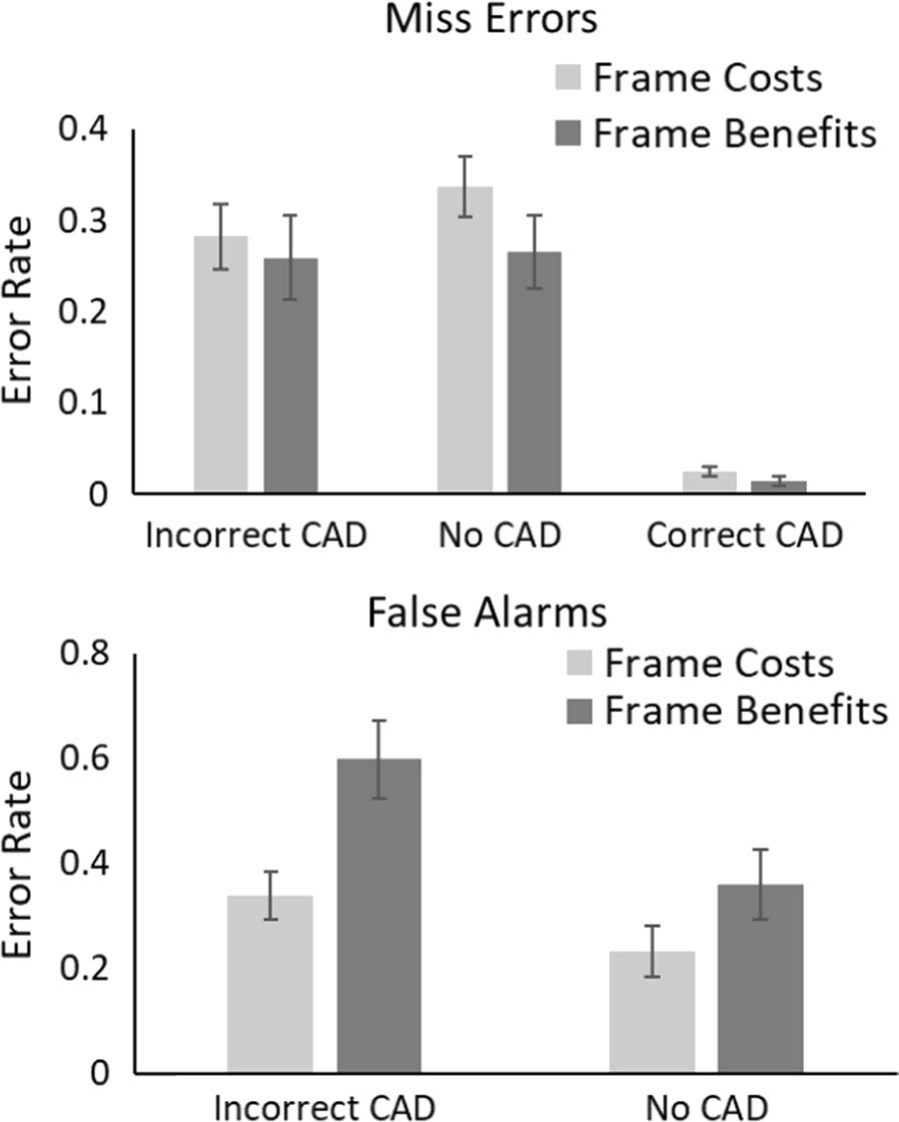
Proportion of miss errors and false alarms in Experiment 3. Error bars represent the standard error

The outlier procedure removed 2.00% of all data. Error rates for all conditions are presented in Fig. 7. The data from one participant were removed from analysis in the Frame Benefits condition because they pressed the ‘m’ key on all trials showing that they were not following task instructions. The remaining data from the 19 participants were entered into analysis (please note that this is still greater than the minimum number of 14 participants, per condition, needed for sufficient power).

#### Miss errors

A 3 × 2 mixed ANOVA on miss errors with within-participant factors of CAD (Correct CAD, Incorrect CAD and No CAD) and between-participant factor of Framing (Benefits vs. Costs) revealed a significant main effect of CAD, *F*(2, 74) = 67.63, *p* < 0.001, *η*_*p*_^2^ = 0.65; there were fewer miss errors in the Correct CAD compared to the Incorrect and No CAD conditions. There was no significant effect of Framing, *F*(1, 37) = 1.15, *p* = 0.29, *η*_*p*_^2^ = 0.03. The CAD × Framing interaction was not significant, *F*(2, 74) = 0.71, *p* = 0.50, *η*_*p*_^2^ = 0.02. As the CAD × Framing interaction was not significant, we did not break this interaction down further.

#### False alarms

A 2 × 2 mixed ANOVA on false alarms with within-participant factors of CAD (No CAD vs. Incorrect CAD) and a between-participant factor of Framing (Benefits vs. Costs) revealed a significant main effect of CAD, *F*(1, 37) = 34.18, *p* < 0.001, *η*_*p*_^2^ = 0.48, in which there were more false alarms with Incorrect CAD compared to No CAD. There was a main effect of Framing, *F*(1, 37) = 5.89, *p* = 0.02, *η*_*p*_^2^ = 0.14, in which there were fewer false alarms in the Frame Costs compared to the Frame Benefits condition. The CAD × Framing Interaction was also significant, *F*(1, 37) = 5.10, *p* = 0.03, *η*_*p*_^2^ = 0.12. Given the CAD × Framing interaction was significant, planned t-tests were used to break-down this interaction. For Incorrect CAD, participants made fewer false alarms in the Frame Costs compared to the Frame Benefits Condition, t(37) = 2.99, *p* = 0.005, d = 0.96, with substantial evidence in support of the alternative, BF_10_ = 8.65. As would be expected, there was no significant difference in false alarms across Framing conditions when there was No CAD, t(37) = 1.53, *p* = 0.13, d = 0.49, with anecdotal evidence in support of the null, BF_10_ = 0.78.

#### Signal detection theory

**Fig. 8:**
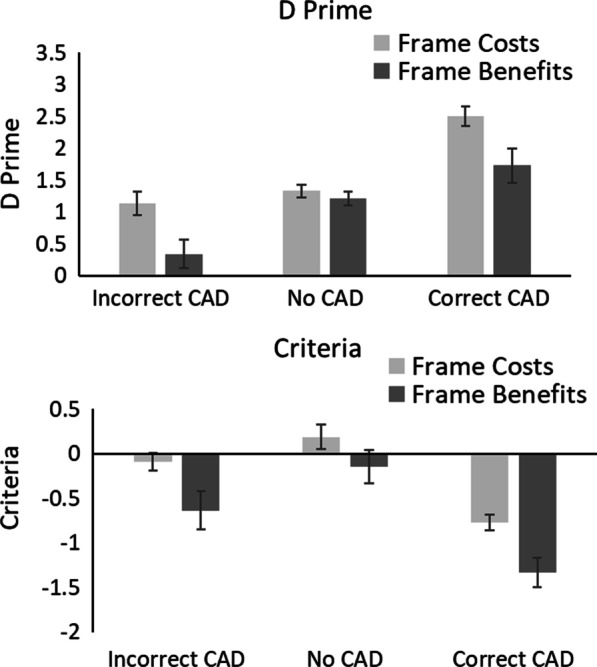
D’ and c values in Experiment 3. Error bars represent the standard error

Figure 8 shows the *d’* and *c* values.

#### Sensitivity (*d*’)

A 3 × 2 mixed ANOVA on *d’* with within-participant factors of CAD (Correct CAD, Incorrect CAD and No CAD) and a between-participant factor of Framing (Benefits vs. Costs) revealed a significant main effect of CAD, *F*(2, 74) = 65.54, *p* < 0.001, *η*_*p*_^2^ = 0.64, in which *d’* was greatest for Correct CAD, followed by No CAD and then Incorrect CAD. There was a significant effect of Framing, *F*(1, 37) = 6.98, *p* = 0.01, *η*_*p*_^2^ = 0.16, in which *d’* was greater in the Frame Costs than the Frame Benefits condition. There was also a significant CAD × Framing interaction, *F*(2, 74) = 5.11, *p* = 0.008, *η*_*p*_^2^ = 0.12. Given the CAD × Framing interaction was significant, planned t-tests were used to break-down this interaction. For Incorrect CAD, *d’* was greater in the Frame costs compared to the Frame Benefits Condition, t(37) = 2.81, *p* = 0.008, d = 0.90, with substantial evidence in support of the alternative, BF_10_ = 6.01. *D’* was also greater for the Frame Costs than the Frame Benefits condition for Correct CAD, t(37) = 2.54, *p* = 0.02, d = 0.82, with substantial evidence in support of the alternative, BF_10_ = 3.61. There was no difference in *d’* across framing conditions when there was No CAD, t(37) = 0.78, *p* = 0.44, d = 0.25, with anecdotal evidence in support of the null, BF_10_ = 0.40.

#### Criteria, (*c*)

A 3 × 2 mixed ANOVA on *c* with within-participant factors of CAD (Correct CAD, Incorrect CAD and No CAD) and between participant factor of Framing (Benefits vs. Costs) revealed a significant main effect of CAD, *F*(2, 74) = 113.11, *p* < 0.001, *η*_*p*_^2^ = 0.75, in which participants were more willing to respond that a mass was present with Correct CAD, followed by Incorrect and then No CAD. There was a significant effect of Framing, *F*(1, 37) = 5.88, *p* = 0.02, *η*_*p*_^2^ = 0.14, in which participants were more willing to respond that a mass was present in the Frame Benefits than the Frame Costs condition. There was no significant CAD × Framing interaction, *F*(2, 74) = 1.55, *p* = 0.22, *η*_*p*_^2^ = 0.04. As the CAD × Framing interaction was non-significant, we did not break this interaction down further.

### Discussion

Experiment 3 determined whether framing the costs of CAD would reduce the over-reliance effect when the target had a low prevalence. The results showed that although miss errors were not affected across conditions, participants made fewer false alarms when they received instructions framing the fallibility of CAD. Examining this further, we see the effect was significant in the Incorrect CAD condition but not in the No CAD condition. Thus, with the knowledge that CAD cues can be incorrect, people were less likely to falsely declare that a target was present when CAD incorrectly indicated the presence of a cancer.

Examining the SDT data, we see that the change in search behaviour with framing occurred due to an overall change in both *d’* and *c*. Framing of CAD fallibility increased both sensitivity and changed people’s response bias so that they were less likely to respond that a target was there. The results also showed that the sensitivity change was affected by CAD so that the ability to detect a target from noise was greater in the Frame Costs condition when CAD cues were incorrect—leading to a decrease in false alarms.

Please note that in Experiment 3 we did not observe a reduction in miss errors for Incorrect CAD conditions (which was observed in Experiment 2). However, within medical screening, as false alarm errors are associated with their own issues (i.e. an increase in anxiety for patients, follow-up invasive medical examinations, increased healthcare costs, etc.) it is also important to minimise these types of errors. Our results show that framing the costs of CAD is an effective way to do this.

## General discussion

This study investigated the effects of CAD on the search for and detection of a cancerous mass in a mammogram. Kunar et al. (2017) suggested that CAD cues produce an over-reliance where people become overly dependent on the technology, leading to issues when the CAD cues are incorrect. The current work determined whether this over-reliance effect could be reduced depending on how the costs or benefits of CAD were framed or whether the mere presence of CAD would always lead to an over-reliance effect.

Experiments 1 and 2 provided people with information framing either the benefits of CAD or the costs of CAD. It was found that a mild warning about the costs/benefits of CAD did little to change search behaviour (Experiment 1). However, giving people a stronger warning and explicitly telling people not to use CAD in Experiment 2 led to an improvement in search performance in terms of a reduction in miss errors and false alarms when the CAD cue was incorrect. Replicating the effect of this strong warning on conditions where the cancer had a lower prevalence showed that although there was no reduction in miss errors, there was again a reduction in false alarms with incorrect CAD (Experiment 3). Interestingly, in all experiments there was still an over-reliance effect of CAD, where miss errors and false alarms were greater when the CAD cue was incorrect.

The results of these experiments demonstrated two important points. First, CAD cues cannot be easily ignored. Even under circumstances of explicit instructions and warnings, participants were still influenced by CAD. This is interesting as it shows how ‘hard-wired’ the visual system and decision processes are so that, even when people know that these cues are hazardous, they cannot fail but to pay attention to them. From the visual attention literature, we know that certain features can capture attention automatically (see Wolfe & Horowitz, 2004, 2017 for a review) and that highly salient, exogenous cues capture attention in a bottom-up manner (e.g. Yantis & Jonides, 1984; Jonides & Yantis, 1988). While there is some evidence to suggest that top-down factors can mitigate bottom-up attention in some cases (e.g. Bacon & Egeth, 1994; Folk et al., 1992), there is also evidence to suggest that some bottom-up signals are immune to top-down control (e.g. Remington et al., 1992; Theeuwes, 2004). More recently, Sawaki and Luck (2010) have suggested a hybrid approach known as the signal suppression hypothesis whereby bottom-up signals from salient distractors can be actively suppressed by top-down processes if they are irrelevant to the task (see also Gaspelin et al., 2015, 2017). Furthermore, people can learn to attentionally suppress regularities in search including the location (Wang & Theeuwes, 2018), temporal properties (Xu et al., 2021) and colours of a salient distractors (Stilwell et al., 2019). This latter research suggests that the role of selection history is important in attention (Awh et al., 2012) and that selection history and value (for example, reward or emotion, Kunar et al., 2014) are thought to modulate attention and activity in the Priority Map (Wolfe, 2021).

In relation to our work, the CAD cues can act as both relevant (in correct CAD trials) and irrelevant (when the CAD cues are incorrect) signals. Thus, the need to suppress CAD priority signals would fluctuate throughout the mammogram reading session. On a trial-by-trial basis, participants had no, a priori, way to determine whether CAD was going to be a relevant (helpful) cue or an irrelevant (distracting) cue that needed to be suppressed. Furthermore, in these experiments the CAD cue was accurate 60% of the time. It could be that readers learned this regularity and used this selection history to weight CAD cues higher on the Priority Map, leading to attentional capture when they were presented. One way to test this possibility would be to run a study in which the CAD cues were 100% inaccurate and so participants never experienced any correct cues. However, from a practical/applied point of view this does not seem sensible because the results would only be applicable if in the real-world CAD cues were ever 100% incorrect—which seems extremely unlikely. Nonetheless, from a theoretical point of view, this possibility might contribute to the apparently stubborn overreliance effect of CAD cues.

Please note that, while in these experiments we used a CAD cue in the form of a salient red box, in clinical settings the form of the CAD can vary (circles of various colours, arrows, highlighted regions etc.). However, all CAD cues will be designed to be clearly visible by the reader and so will, by definition, have some form of ‘attention-grabbing’ bottom-up signal. Indeed, if a CAD cue was not easy to find or did not draw attention, then a reader would need to perform two difficult search tasks, one to find the CAD cue and another to find a possible non-cued mass—rendering the cue ineffective. Therefore, it would be sensible to suggest that the attentional capture exhibited in our studies is generalisable to those CAD systems that use exogenous CAD prompts in different forms. It will be up to future research to investigate this further.

Second, although an over-reliance effect was witnessed across all experiments and never fully disappeared, framing of the CAD system did *reduce* the dependency on the technology. Giving people strong warnings and instruction sets about the costs of CAD led to a reduction in false alarms at both high prevalence and low prevalence (Experiments 2 and 3) and a reduction in miss errors at high prevalence (Experiment 2). Of course, in mammography, prevalence rates of a cancer are much lower than 50%; therefore, prior knowledge may not be beneficial to the reduction in miss errors in a clinical setting. Miss errors at LP are thought to occur in response to a Multiple Decision Model (MDM) where (i) the search quitting threshold is reduced so less time is spent searching the displays and (ii) there is a shift in response bias, so that observers are less willing to respond that a target is present (Wolfe & Van Wert, 2010). Both of these factors lead to large proportions of miss errors at LP that are difficult to alleviate (Wolfe et al., 2007). From our work, it seems that the effect of framing the costs of CAD is not enough to counter the high miss errors observed in LP search and proposed by the MDM.

However, framing does reduce the over-reliance effect in terms of lowering false alarms. False alarms are important to reduce in a clinical setting to avoid unnecessary costs, worry to patients and invasive treatments of further medical tests. If prior knowledge of CAD can alleviate at least some of these burdens for patients, then it is a worthwhile area of research. The findings are especially important given that investment into CAD, AI and its development has been on the rise, yet little research has been conducted investigating questions such as how to best present and regulate AI to readers and healthcare professionals (Reddy et al., 2020). For present purposes, our research has shown that with a few simple, low-cost instructions the reading performance of humans using CAD can dramatically improve.

One obvious limitation of our research is the use of non-medical observers as readers. Without medical expertise, our participants may have been more vulnerable to over-reliance effects of CAD compared to trained clinicians. Despite this, there is some evidence that radiologists show over-reliance effects of CAD in clinical settings. In a study in which eight radiologists searched mammograms for anomalies using CAD, Zheng et al. (2004) showed that fewer abnormalities were detected if they appeared in non-cued areas. Hupse et al. (2013) also found that the way radiologists interacted with CAD varied depending on how much experience they had, with more experienced readers being less reliant on CAD prompts. On this basis, it might be that framing information about the costs/benefits of CAD would affect experienced radiologists and newly trained radiologists differently, with trainee radiologists benefitting the most from knowledge of CAD fallibility. Determining if this is the case will be a useful goal for future research.

In the current experiments, participants read mammograms where a CAD system was always employed. Therefore, it was not possible to determine how cancer detection compared to performance in conditions in which a CAD system was never presented. Although it was not practical to test this condition in this paper, other researchers have compared error rates in conditions that used a CAD system, to error rates in conditions that never presented CAD (Drew et al., 2020; Kunar et al., 2017). The results suggest that the over-reliance on CAD led to more miss errors when a CAD system was incorrect in comparison to performance when a CAD system was never presented (see Phelps et al., 2021, for example of over-reliance with other medical images). Given that the use of CAD in medical screening is becoming more prevalent as technology evolves, it is vitally important to work out how presentation of these CAD cues affects decisions in clinical settings, in order to mitigate any effects of over-dependence.

The findings that people use CAD cues when explicitly told not to are also important for CAD developers. Adding a salient CAD cue into a search may act against a radiologist’s normal search process. Not only do CAD cues capture attention, but their presence may affect holistic or global processing, which has been found to be important in mammography (e.g. Evans et al., 2013a, 2013b; Drew et al., 2013; Kundel et al., 2007). Other research has suggested CAD would be more effective as a simpler system where, instead of presenting a cue on the image, CAD simply gives a ‘yes/no’ indication that a cancer may be present (e.g. Goldenberg & Peled, 2011). Using CAD in this manner may be more practical in an applied setting if it reduces factors such as attention capture. Further research would be needed to investigate this.

In accounting for our findings, we have emphasised the influence of CAD cues on the search process itself. For example, we have noted that the CAD cue likely attracts attention and influences a participant’s search pattern. That is, the cue might cause an area to be prioritised for search with a consequent reduction in the extent to which other areas are searched. This might lead to a miss error when the cue is incorrect or facilitated detection when it is correct. However, it is important to note that there are at least two main processes at work here—a search stage and a decision stage. First, a relevant mass must be detected (the search stage); then, once a *potential* mass is found, a decision has to be made as to whether it is indeed a mass, and if so whether it is benign or cancerous. With this in mind, in Experiment 1, cancers were more likely to be mis-identified as benign masses when an incorrect CAD cue was present. Here the CAD cues appeared to have also modulated the decision process even after a mass had been found. Although this question was not the focus of our work, it is another important consideration in determining how CAD affects cancer detection. Please note we have found similar results in some experiments (e.g. Kunar et al., 2017), but not in all experiments from our laboratory. Therefore, it will be up to future experiments to examine how and when CAD affects the decision stage as well as considering whether a mass is missed or not.

Of final note, the work in the paper has implications for how companies designing CAD algorithms should market their product. It is probable that most CAD marketing campaigns will focus on the benefits of the technology (i.e. very few companies will be likely to highlight the inaccurate parts of their algorithms). This could be dangerous to readers in breast-screening practices, who are investing in this technology and are under the assumption that the addition of CAD will only be beneficial. Instead, we propose that healthcare professionals should be made explicitly aware of the costs of CAD and be given training to highlight the instances that CAD can be incorrect. On a positive note, giving people warnings about CAD does little to affect miss errors when the CAD cue is *correct*. CAD algorithms are trained and programmed so that they are likely to be accurate on the majority of trials (although actual accuracy varies across manufacturers). Therefore, it is good to know that giving people warnings about the downsides of CAD still maintains the benefits of CAD by reducing miss errors when the CAD cue is correct, but importantly reduces people’s over-reliance on the technology in instances when CAD is fallible.

## Conclusions

There has been increased investment into AI and CAD as a decision aid in mammography. However, little research has investigated the optimal way to present this technology to human readers. It is imperative that alongside the increased financial investment in AI in medical screening, it is also important to investigate other optimal ways to present this technology to humans for successful interaction. The above research shows that giving non-expert observers knowledge of CAD performance benefits mammogram reading by mitigating the costs of becoming over-dependent on the technology, while still retaining the benefits when the technology detects a cancer. This is a start in understanding how humans interact with automated decision guidance systems; however, these effects also need to be replicated with clinician observers. Elmore and Lee (2022) suggest that the interaction between human reader and CAD is complex and is most likely prone to bias. Furthermore, Masud et al. (2019) have found that there is a lack of research investigating how to best implement CAD in a clinical setting with knowledge gaps in the literature and concerns about radiologists' confidence level in the technology (see also Jungmann et al., 2021). To understand these interactions and to move forward in the field, we need to better evaluate how humans interact with CAD in clinical settings and also start a more rigorous dialogue between those that develop CAD with those healthcare professionals that will ultimately end up using it.

## Data Availability

The datasets used and/or analysed during the current study are available from the corresponding author on reasonable request. The datasets generated and/or analysed during the current study are available in the Open Science Framework repository, https://osf.io/m832p/.

## Abbreviations

LP: Low prevalence
CAD: Computer-Aided Detection
ANOVA: Analysis of variance
RTs: Reaction times
UK: United Kingdom
USA: United States of America
MDM: Multiple decision model

## Footnotes

1 Please note that 10% target prevalence rate is still higher than is typically observed in a clinical setting; however, it is a prevalence rate that has often been used in laboratory studies to investigate the LP Effect (e.g. Navalpakkam et al., 2009; Peltier & Becker, 2016).

2 Please note there was no Mass as a factor as False Alarms are only possible on target absent trials.

3 False alarm or miss error rates of 0 and 1 were adjusted using the formulas 1/2*n* and 1 − (1/2*n*), where *n* = the number of trials (Macmillan & Kaplan, 1985, see also Russell & Kunar, 2012; Wolfe et al., 2007; Kunar et al., 2021, who used this procedure).

## References

[CR1] Aro AR (2000). False-positive findings in mammography screening induces short-term distress: Breast cancer-specific concern prevails longer. European Journal of Cancer.

[CR2] Awh E, Belopolsky A, Theeuwes J (2012). Top-down versus bottom-up attentional control: A failed theoretical dichotomy. Trends in Cognitive Sciences.

[CR3] Azavedo E, Zackrisson S, Mejàre I, Heibert Arnlind M (2012). Is single reading with computer-aided detection (CAD) as good as double reading in mammography screening? A systematic review. BMC Medical Imaging.

[CR4] Bacon WF, Egeth HE (1994). Overriding stimulus-driven attentional capture. Perception & Psychophysics.

[CR5] Bennett RL, Blanks RG, Moss SM (2006). Does the accuracy of single reading with CAD (computer-aided detection) compare with that of double reading? A review of the literature. Clinical Radiology.

[CR6] Castellino RA (2005). Computer aided detection (CAD): An overview. Cancer Imaging.

[CR7] Cox PH, Kravitz DJ, Mitroff SR (2021). Great expectations: Minor differences in initial instructions have a major impact on visual search in the absence of feedback. Cognitive Research.

[CR8] Drew T, Cunningham C, Wolfe JM (2012). When and why might a Computer Aided Detection (CAD) system interfere with visual search? An eye-tracking study. Academic Radiology.

[CR9] Drew T, Evans K, Võ MLH, Jacobson FL, Wolfe JM (2013). Informatics in radiology: what can you see in a single glance and how might this guide visual search in medical images?. Radiographics.

[CR10] Drew T, Guthrie J, Reback I (2020). Worse in real life: An eye-tracking examination of the cost of CAD at low prevalence. Journal of Experimental Psychology: Applied.

[CR11] Elmore JG, Lee CI (2022). Artificial intelligence in medical imaging-learning from past mistakes in mammography. JAMA Health Forum.

[CR12] Evans KK, Birdwell RL, Wolfe JM (2013). If you don’t find it often, you often don’t find it: Why some cancers are missed in breast cancer screening. PLoS ONE.

[CR13] Evans KK, Georgian-Smith D, Tambouret R, Birdwell RL, Wolfe JM (2013). The gist of the abnormal: Above-chance medical decision making in the blink of an eye. Psychonomic Bulletin & Review.

[CR15] Faul F, Erdfelder E, Lang A-G, Buchner A (2007). G*Power 3: A flexible statistical power analysis program for the social, behavioral, and biomedical sciences. Behavior Research Methods.

[CR16] Fleck MS, Mitroff SR (2007). Rare targets are rarely missed in correctable search. Psychological Science.

[CR17] Folk CL, Remington RW, Johnston JC (1992). Involuntary covert orienting is contingent on attentional control settings. Journal of Experimental Psychology: Human Perception and Performance.

[CR18] Gaspelin N, Leonard CJ, Luck SJ (2015). Direct evidence for active suppression of salient-but-irrelevant sensory inputs. Psychological Science.

[CR19] Gaspelin N, Leonard CJ, Luck SJ (2017). Suppression of overt attentional capture by salient-but-irrelevant color singletons. Attention, Perception, Psychophysics.

[CR20] Gilbert FJ, Astley SM, Gillan MG, Agbaje OF, Wallis MG, James J, Boggis CR, Duffy SW (2008). the CADET II group: Single reading with computer-aided detection for screening mammography. New England Journal of Medicine.

[CR21] Goldenberg R, Peled N (2011). Computer-aided simple triage. International Journal of Computer Assisted Radiology and Surgery.

[CR22] Green DM, Swets JA (1967). Signal detection theory and psychophysics.

[CR23] Guerriero C, Gillan MGC, Cairns J, Wallis MG, Gilbert FJ (2011). Is computer aided detection (CAD) cost effective in screening mammography? A model based on the CADET II study. BMC Health Services Research.

[CR24] Gur D, Rockette HE, Armfield DR, Blachar A, Bogan JK, Brancatelli G, Britton CA, Brown ML, Davis PL, Ferris JV, Fuhrman CR (2003). Prevalence effect in a laboratory environment. Radiology.

[CR25] Harvey H, Karpati E, Khara G, Korkinof D, Ng A, Austin C, Rijken T, Kecskemethy P (2019). The role of deep learning in breast screening. Current Breast Cancer Reports.

[CR26] Heath, M., Bowyer, K., Kopans, D., Kegelmeyer, W. P., Moore, R., Chang, K., & MunishKumaran, S. (1998) Digital mammography. In *Proceedings of the fourth international workshop on digital mammography* (pp. 457–460). Kluwer Academic Publishers.

[CR27] Heath, M., Bowyer, K., Kopans, D., Moore, R., & Kegelmeyer, W. P. (2001). In M. J. Yaffe (Ed.) *Proceedings of the fifth international workshop on digital mammography* (pp. 212–218). Medical Physics Publishing.

[CR28] Henriksen EL, Carlsen JF, Vejborg IM, Nielsen MB, Lauridsen CA (2019). The efficacy of using computer-aided detection (CAD) for detection of breast cancer in mammography screening: A systematic review. Acta Radiologica.

[CR29] Horowitz TS (2017). Prevalence in visual search: From the clinic to the lab and back again. Japanese Psychological Research.

[CR30] Houssami N, Given-Wilson R, Ciatto S (2009). Early detection of breast cancer: Overview of the evidence on computer-aided detection in mammography screening. Journal of Medical Imaging and Radiation Oncology.

[CR31] Hupse R, Samulski M, Lobbes MB, Mann RM, Mus R, den Heeten GJ, Beijerinck D, Pijnappel RM, Boetes C, Karssemeijer N (2013). Computer-aided detection of masses at mammography: Interactive decision support versus prompts. Radiology.

[CR32] James JJ, Gilbert FJ, Wallis MG, Gillan MG, Astley SM, Boggis CR, Agbaje OF, Brentnall AR, Duffy SW (2010). Mammographic features of breast cancers at single reading with computer-aided detection and at double reading in a large multicenter prospective trial of computer-aided detection: CADET II. Radiology.

[CR33] Jarosz AF, Wiley J (2014). What are the odds? A practical guide to computing and reporting Bayes factors. The Journal of Problem Solving.

[CR34] JASP Team. (2021). JASP (Version 0.16) [Computer software].

[CR35] Jeffreys H (1961). Theory of probability.

[CR36] Jonides J, Yantis S (1988). Uniqueness of abrupt visual onset in capturing attention. Perception & Psychophysics.

[CR37] Julesz B (1981). A theory of preattentive texture discrimination based on first order statistics of textons. Biology and Cybernetics.

[CR38] Jungmann F, Jorg T, Hahn F, Dos Santos DP, Jungmann SM, Düber C, Mildenberger P, Kloeckner R (2021). Attitudes toward artificial intelligence among radiologists, IT specialists, and industry. Academic Radiology.

[CR39] Kahn BE, Luce MF (2003). Understanding high-stakes consumer decisions: Mammography adherence following false-alarm test results. Marketing Science.

[CR40] Kassin S, Dror I, Kukucka J (2013). The forensic confirmation bias: Problems, perspectives, and proposed solutions. Journal of Applied Research in Memory and Cognition.

[CR41] Keen JD, Keen JM, Keen JE (2018). Utilization of computer-aided detection for digital screening mammography in the United States, 2008 to 2016. Journal of the American College of Radiology.

[CR42] Kunar MA (2022). The optimal use of computer aided detection to find low prevalence cancers. Cognitive Research: Principles and Implications.

[CR43] Kunar MA, Rich AN, Wolfe JM (2010). Spatial and temporal separation fails to counteract the effects of low prevalence in visual search. Visual Cognition.

[CR44] Kunar MA, Watson DG, Cole L, Cox A (2014). Negative emotional stimuli reduce contextual cueing but not response times in inefficient search. The Quarterly Journal of Experimental Psychology.

[CR45] Kunar MA, Watson DG, Taylor-Phillips S (2021). Double reading reduces miss errors in low prevalence search. Journal of Experimental Psychology: Applied.

[CR46] Kunar MA, Watson DG, Taylor-Phillips S, Wolska J (2017). Low prevalence search for cancers in mammograms: Evidence using laboratory experiments and computer aided detection. Journal of Experimental Psychology: Applied.

[CR47] Kundel HL, Nodine CF, Conant EF, Weinstein SP (2007). Holistic component of image perception in mammogram interpretation: Gaze-tracking study. Radiology.

[CR48] Lehman CD, Wellman RD, Buist DSM, Kerlikowske K, Tosteson ANA, Miglioretti DL (2015). Diagnostic accuracy of digital screening mammography with and without computer-aided detection. JAMA Internal Medicine.

[CR49] Luck SJ, Gaspelin N, Folk CL, Remington RW, Theeuwes J (2021). Progress toward resolving the attentional capture debate. Visual Cognition.

[CR50] Macmillan, N. A., & Creelman, C. D. (2005). *Detection theory: A user's guide*, 2nd Edn, Cambridge University Press.

[CR51] Macmillan NA, Kaplan HL (1985). Detection theory analysis of group data: estimating sensitivity from average hit and false-alarm rates. Psychological Bulletin.

[CR52] Madrid J, Hout MC (2019). Examining the effects of passive and active strategies on behavior during hybrid visual memory search: Evidence from eye tracking. Cognitive Research.

[CR53] Masud R, Al-Rei M, Lokker C (2019). Computer-aided detection for breast cancer screening in clinical settings: Scoping review. JMIR Medical Informatics.

[CR54] Mitroff SR, Biggs AT (2014). The ultra-rare-item effect: Visual search for exceedingly rare items is highly susceptible to error. Psychological Science.

[CR55] Navalpakkam V, Koch C, Perona P (2009). Homo economicus in visual search. Journal of Vision.

[CR56] Obenauer S, Sohns C, Werner C, Grabbe E (2006). Impact of breast density on computer-aided detection in full-field digital mammography. Journal of digital imaging.

[CR57] Peltier C, Becker MW (2016). Decision processes in visual search as a function of target prevalence. Journal of Experimental Psychology: Human Perception and Performance.

[CR58] Phelps EE, Wellings R, Griffiths F, Hutchinson C, Kunar M (2017). Do medical images aid understanding and recall of medical information? An experimental study comparing the experience of viewing no image, a 2D medical image and a 3D medical image alongside a diagnosis. Patient Education and Counseling.

[CR59] Phelps EE, Wellings R, Kunar M, Hutchinson C, Griffiths F (2021). A qualitative study exploring the experience of viewing three-dimensional medical images during an orthopaedic outpatient consultation from the perspective of patients, health care professionals, and lay representatives. Journal of Evaluation in Clinical Practice.

[CR60] Reddy S, Allan S, Coghlan S, Cooper P (2020). A governance model for the application of AI in health care. Journal of the American Medical Informatics Association.

[CR61] Remington RW, Johnston JC, Yantis S (1992). Involuntary attentional capture by abrupt onsets. Perception & Psychophysics.

[CR62] Rich AN, Kunar MA, Van Wert MJ, Hidalgo-Sotelo B, Horowitz TS, Wolfe JM (2008). Why do we miss rare targets? Exploring the boundaries of the low prevalence effect. Journal of Vision.

[CR63] Russell N, Kunar MA (2012). Color and spatial cueing in low prevalence visual search. The Quarterly Journal of Experimental Psychology.

[CR64] Samulski M, Hupse R, Boetes C, Mus R, Heeten G, Karssemeijer N (2010). Using computer-aided detection in mammography as a decision support. European Radiology.

[CR65] Sato M, Kawai M, Nishino Y, Shibuya D, Ohuchi N, Ishibashi T (2014). Cost-effectiveness analysis for breast cancer screening: Double reading versus single + CAD reading. Breast Cancer (Tokyo, Japan).

[CR66] Sawaki R, Luck SJ (2010). Capture versus suppression of attention by salient singletons: Electrophysiological evidence for an automatic attend-to-me signal. Attention, Perception & Psychophysics.

[CR67] Sellier AL, Scopelliti I, Morewedge CK (2019). Debiasing training improves decision making in the field. Psychological Science.

[CR68] Sibly. (2004). BlitzMax (Version 1.48) [Computer software].

[CR69] Soo MS, Rosen EL, Xia JQ, Ghate S, Baker JA (2005). Computer-aided detection of amorphous calcifications. American Journal of Roentgenology.

[CR70] Stilwell BT, Bahle B, Vecera SP (2019). Feature-based statistical regularities of distractors modulate attentional capture. Journal of Experimental Psychology: Human Perception and Performance.

[CR71] Taylor P, Potts HW (2008). Computer aids and human second reading as interventions in screening mammography: Two systematic reviews to compare effects on cancer detection and recall rate. European Journal of Cancer.

[CR72] Thaler RH, Sunstein CR (2008). Nudge: Improving decisions about health, wealth, and happiness.

[CR73] Theeuwes J (1994). Stimulus-driven capture and attentional set: Selective search for color and visual abrupt onsets. Journal of Experimental Psychology: Human Perception and Performance.

[CR74] Theeuwes J (2004). Top-down search strategies cannot override attentional capture. Psychonomic Bulletin & Review.

[CR75] Van Wert MJ, Horowitz TS, Wolfe JM (2009). Even in correctable search, some types of rare targets are frequently missed. Attention, Perception & Psychophysics.

[CR76] Wagenmakers EJ, Love J, Marsman M, Jamil T, Ly A, Verhagen J, Selker R, Gronau QF, Dropmann D, Boutin B, Meerhoff F (2018). Bayesian inference for psychology. Part II: Example applications with JASP. Psychonomic Bulletin & Review.

[CR77] Wagenmakers E-J, Marsman M, Jamil T, Ly A, Verhagen AJ, Love J, Selker R, Gronau QF, Šmíra M, Epskamp S, Matzke D, Rouder JN, Morey RD (2018). Bayesian inference for psychology. Part I: Theoretical advantages and practical ramifications. Psychonomic Bulletin & Review.

[CR78] Wang B, Theeuwes J (2018). Statistical regularities modulate attentional capture. Journal of Experimental Psychology: Human Perception and Performance.

[CR79] Wolfe JM (2021). Guided search 6.0: An updated model of visual search. Psychonomic Bulletin and Review.

[CR80] Wolfe J, Horowitz T (2004). What attributes guide the deployment of visual attention and how do they do it? Nature reviews. Neuroscience.

[CR81] Wolfe J, Horowitz T (2017). Five factors that guide attention in visual search. Nature Human Behaviour.

[CR82] Wolfe JM, Horowitz TS, Kenner NM (2005). Rare items often missed in visual search. Nature.

[CR83] Wolfe JM, Horowitz TS, Ven Wert MJ, Kenner NM, Place SS, Kibbi N (2007). Low target prevalence is a stubborn source of errors in visual search tasks. Journal of Experimental Psychology.

[CR84] Wolfe JM, VanWert MJ (2010). Varying target prevalence reveals two, dissociable decision criteria in visual search. Current Biology.

[CR85] Xu Z, Los SA, Theeuwes J (2021). Attentional suppression in time and space. Journal of Experimental Psychology: Human Perception and Performance.

[CR86] Yantis S, Jonides J (1984). Abrupt visual onsets and selective attention: Evidence from visual search. Journal of Experimental Psychology: Human Perception & Performance.

[CR87] Zheng B, Swensson RG, Golla S, Hakim CM, Shah R, Wallace L, Gur D (2004). Detection and classification performance levels of mammographic masses under different computer-aided detection cueing environments. Academic Radiology.

